# Random mutagenesis and phage display technology as a tool for identifying ige epitopes of the birch pollen allergen Bet v 1

**DOI:** 10.1186/2045-7022-4-S2-O5

**Published:** 2014-03-17

**Authors:** Elisabeth Eva Guhsl, Gerlinde Hofstetter, Christof Ebner, Wolfgang Hemmer, Heimo Breiteneder, Christian Radauer

**Affiliations:** 1 Medical University of Vienna, Department of Pathophysiology and Allergy Research, Vienna, Austria; 2 Ambulatory for Allergy and Clinical Immunology, Vienna, Austria; 3 Floridsdorf Allergy Center, Vienna, Austria

## Background

IgE-binding epitopes of the major birch pollen allergen Bet v 1 are dependent on the native structure of the allergen. To date, only a single IgE epitope has been identified. We aimed to identify additional epitopes by directed *in vitro* evolution of non-IgE-binding structural homologues to proteins carrying single IgE-binding patches. For that purpose, we chose cytokinin-specific binding protein (CSBP) from *Vigna radiata* (mung bean), a structural homologue of Bet v 1 with only 31% sequence identity, as a template subjected to a combination of random mutagenesis and phage display.

## Methods

CSBP was expressed in *Escherichia coli* and purified by chromatographic methods. The protein integrity was verified by SDS-PAGE, mass spectrometry and circular dichroism and IgE binding assessed by ELISA using sera from a panel of birch pollen allergic patients. Random mutagenesis of CSBP was performed by PCR using mutagenic nucleotide analogues. Phage display libraries of randomly mutated CSBP were created in the filamentous phage M13 by inserting PCR products into the phagmid pTP127. Biopanning was performed for selecting phages with binding activity to birch pollen allergic patients' IgE. After each panning round, an additional mutagenesis was performed and a new library created. IgE binding activities of enriched phages and of bacterial colonies representing single clones were analysed by transfer to nitrocellulose and immunostaining.

## Results

Purified recombinant CSBP revealed a secondary structure with high similarity to that of Bet v 1, but only low IgE binding in 11 of 35 sera from Bet v 1-sensitised birch pollen allergic patients. Phage libraries CSBPm1, CSBPm2 and CSBPm3 with diversities of 10^5^-10^7^ different clones were created. Sequencing of 19 randomly picked clones from the unselected CSBPm1 library showed an average mutation rate of 5% (range 2.5-11.5). Analysis of single clones from the first two panning rounds yielded clones which expressed IgE binding mutant proteins.

## Conclusion

Directed *in vitro* evolution of CSBP by random mutagenesis of surface residues might be a suitable tool for defining conformational IgE binding epitopes of the birch pollen allergen Bet v 1.

This study was supported by grant P-22559-B11 from the Austrian Science Fund.

